# Eradication of HIV by Transplantation of CCR5-Deficient Hematopoietic Stem Cells

**DOI:** 10.1100/tsw.2011.102

**Published:** 2011-05-05

**Authors:** Gero Hütter, Susanne Ganepola

**Affiliations:** ^1^ Institute of Transfusion Medicine and Immunology, Medical Faculty Mannheim, Heidelberg University, Germany; ^2^ German Red Cross Blood Service Baden-Württemberg – Hessen, Germany; ^3^ II Medical Department, Asklepios Klinik Altona, Hamburg, Germany

**Keywords:** HIV, CCR5, CCR5-delta32, allogeneic stem cells, transplantation

## Abstract

Today, 30 years after the onset of the HIV pandemic, although treatment strategies have considerably improved, there is still no cure for the disease. Recently, we described a successful hematopoietic stem cell transplantation in an HIV-1–infected patient, transferring donor-derived cells with a natural resistance against HIV infection. These hematopoietic stem cells engrafted, proliferated, and differentiated into mature myeloid and lymphoid cells. To date, the patient has not required any antiretroviral treatment, more than 4 years after allogeneic transplantation. In the analysis of peripheral blood cells and different tissue samples, including gut, liver, and brain, no viral load or proviral DNA could be detected. Our report raises the hope for further targeted treatment strategies against HIV and represents a successful personalized treatment with allogeneic stem cells carrying a beneficial gene. However, this case has ignited a controversy regarding the question of whether this patient has achieved complete eradication of HIV or not. Here we give an update on open questions, unsolved aspects, and clinical consequences concerning this unique case.

## INTRODUCTION

For cell entry, the human immunodeficiency virus type 1 (HIV-1) uses the highly conserved host elements of both CD4 and the CC chemokine receptor type 5 (CCR5). The fusion of virion and host cell is mediated by forming a six-helix bundle of gp41, bringing the viral and host cell membrane together[1].

In 1996, 13 years after the first description of HIV-1 as the causative agent for the acquired immunodeficiency syndrome (AIDS), several research groups discovered the most important reason of natural protection against HIV transmission. This resistance is based on a frequently occurring mutation in the CCR5 gene leading to a 32-base-pair deletion (CCR5-delta32), introducing a premature stop-codon into the chemokine-receptor locus[2].

Homozygosity for the CCR5-delta32 deletion is associated with a high, but not complete, HIV-1 resistance[3]. Under certain conditions, HIV-1 is able to use CXCR4 as an alternative chemokine coreceptor instead of CCR5. The responsible mutation occurs at V3 of the HIV-1 genome encoding an envelope glycoprotein, binding either to CCR5 (R5 type) or CXCR4 (X4 type)[4]. The switch from R5 to X4 commonly occurs during the natural course of infection and most patients with HIV harbor a significant proportion of X4 quasispecies[5].

Because HIV-1 requires CD4 and predominantly CCR5 for cell entry, this gene and the CCR5-delta32 deletion are the most promising candidates for therapeutic interventions. While CCR5 is not required for viability, the absence of CD4 or CXCR4 is incompatible with human life. Therefore, CCR5 could play a role as the Achilles' heel in HIV-1 infection.

## THE VISION OF TRANSPLANTING CCR5-DEFICIENT CELLS IN PATIENTS WITH HIV INFECTION

The most profound impact on the human immune system is the transplantation of allogeneic hematopoietic stem cells (HSCT) in patients with malignancies. HSCT has become a standard procedure for different types of malignant and nonmalignant diseases, and is also feasible in patients with HIV infection. Initially, there was optimism that transplantation of a new and healthy immune system in patients with HIV might improve the course of infection, but neither allogeneic nor autologous HSCT alone were sufficient to get rid of the virus[6].

Nevertheless, it seemed likely that a CCR5-delta32–based, stem cell–derived approach could improve the course of HIV infection and, in 2001, R. Chow, founder of StemCyte, Inc., applied for a patent to screen allogeneic stem cell donors for a beneficial gene with which to treat HIV infection (U.S. patent 2003/0099621 A1). Chows' group built a database with over 10,000 cord blood units, genotyped for the CCR5-delta32 deletion[7]. However, to date, they have not found appropriate HLA-matched candidates with HIV-1 infection requiring HSCT from their cord blood units.

Compelled from the difficulties in finding probable CCR5-deficient donors, attempts to mimic CCR5 knockdown by gene therapy are particularly attractive, primarily because they block the very first step in HIV-1 infection rather than preventing an event after the establishment of the proviral DNA in the human genome.

Currently, a choice of techniques inhibiting CCR5 expression is available, such as siRNA, short hairpin RNA, and, most promising, zinc-finger nucleases (ZFNs). ZFNs are artificial restriction enzymes generated by fusing a zinc-finger DNA-binding domain to a DNA-cleavage domain. They can be designed to bind a specific genomic DNA and to affect the knockdown of the CCR5 gene, by generating a targeted double-strand break[8].

## THE FIRST ALLOGENEIC TRANSPLANTATION OF STEM CELLS LACKING THE CCR5 RECEPTOR IN A PATIENT WITH HIV INFECTION

In February 2007, 11 years after the first description of the CCR5-delta32 mutation, a medical team of the Charité Berlin performed an allogeneic HSCT in an HIV-infected patient suffering from acute myeloid leukemia using progenitor cells from a donor especially selected to be homozygous for the CCR5-delta32 deletion[9]. Allogeneic HSCT has become a standard treatment for patients with high-risk leukemia, or relapsed and refractory lymphoma, and in 20%, the donor is an HLA-matched sibling. Patients without HLA-matched siblings receive their stem cells from HLA-matched, but unrelated, donors. Currently, there are more than 16 million potential donors registered worldwide and the probability of finding a perfect match is over 90–95%.

During the transplant procedure, the patient received FLAMSA regimen and a graft containing $2.3 \times 10^6$ $CD34^+$ cells/kg body weight. Prophylaxis of Graft vs. host disease (GvHD) consisted of antithymocyte globulin 0.5 mg/kg on day –3 and 2.5 mg/kg on days –2 and –1. Cyclosporine A 2.5 mg/kg/day was given since day –1 and mycophenolate mofetil $3 \times 1$ g/day was started 6 h after transplantation. By using the homozygous CCR5-delta32 stem cells, the patient's CCR5 allele genotype changed as expected. After a relapse of the leukemia on day +332, the patient was treated with a reinduction therapy with cytarabine (100 mg/sqm, days 1–7 and gemtuzumab 6 mg/sqm, days 1+7) leading to a complete remission. On day +391, the patient received total body irradiation with 2 Gy followed by a transplantation of $2.1 \times 10^6$ $CD34^+$ cells/kg body weight from the same CCR5-depleted donor of the first transplantation.

Finally, HIV remained undetectable for more than 4 years after discontinuing antiretroviral therapy after the first transplantation, as determined by viral RNA and proviral DNA PCR assays of peripheral blood, bone marrow, and several tissue biopsies, including the gut and brain (Fig. 1). Interestingly, although antiretroviral medication was stopped 1 day before reinfusion of stem cells and proviral DNA was detectable in peripheral blood mononuclear cells for 60 days after this time point, no viral rebound was observed. Moreover, there was a discrepancy of residual CCR5 expression in different anatomical compartments. Whereas the proportion of CD4+/CCR5+ T cells in the peripheral blood declined from month to month, CCR5-expression macrophages in the gut were still detectable half a year after transplantation (Fig. 2). Finally, during a follow-up period of 4 years, even these residual CCR5-expressing macrophages as a potential reservoir for latently infected cells became undetectable in certain tissue biopsies of the gut as determined by immunofluorescence histology[10].

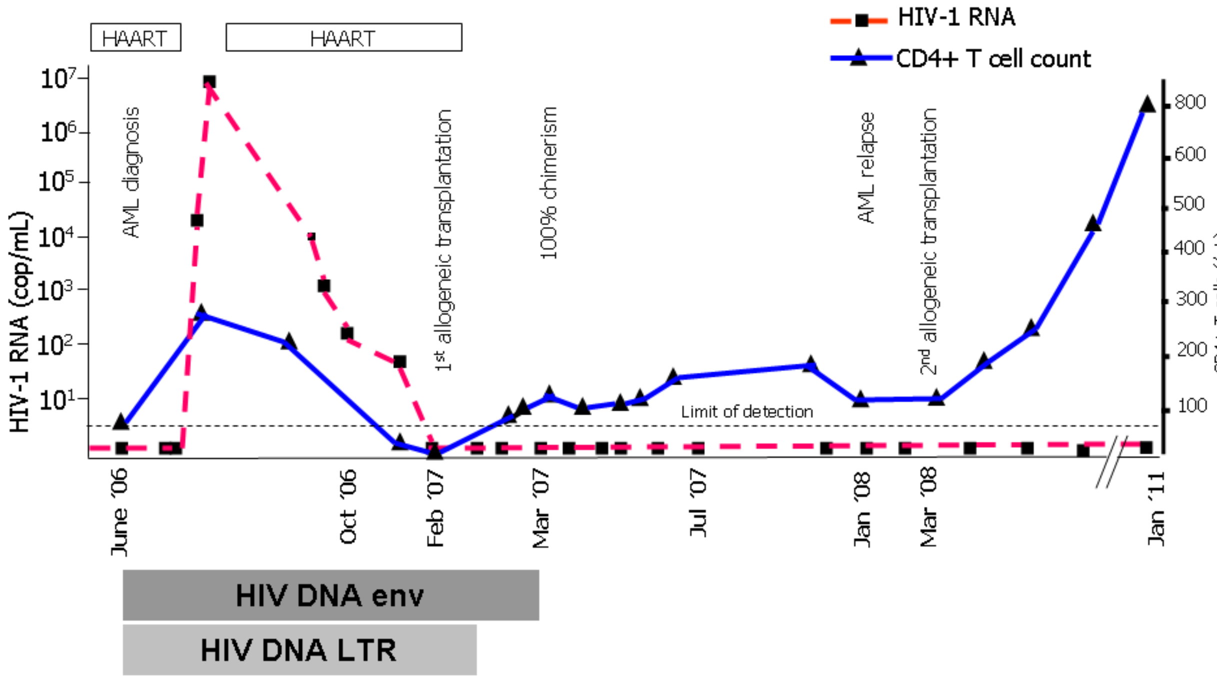

**FIGURE 1.** The clinical course and treatment of leukemia and HIV infection. Measurement of HIV-1 viremia by RNA-PCR (red line) and DNA PCR assays are displayed. HIV-1 RNA could not be detected in peripheral blood and bone marrow during the follow-up since HAART had been discontinued on day –1 up to date. The count of CD4+ T cells in the peripheral blood are displayed as the blue line.

Despite the successful treatment of the patient's leukemia and the long-term control of HIV infection, there have been several setbacks in this case. First, the early relapse of leukemia within the first year after allogeneic transplantation; second, a long period of infections, GvHD reactions of the liver, and other aggravating circumstances; third, and one of the most impairing events, a period of fever, dizziness, disturbance of the consciousness, and loss of the short-term memory after the second transplantation. CT and MRT scans revealed an affection of the white matter of the brain, but all attempts to find a diagnosis by

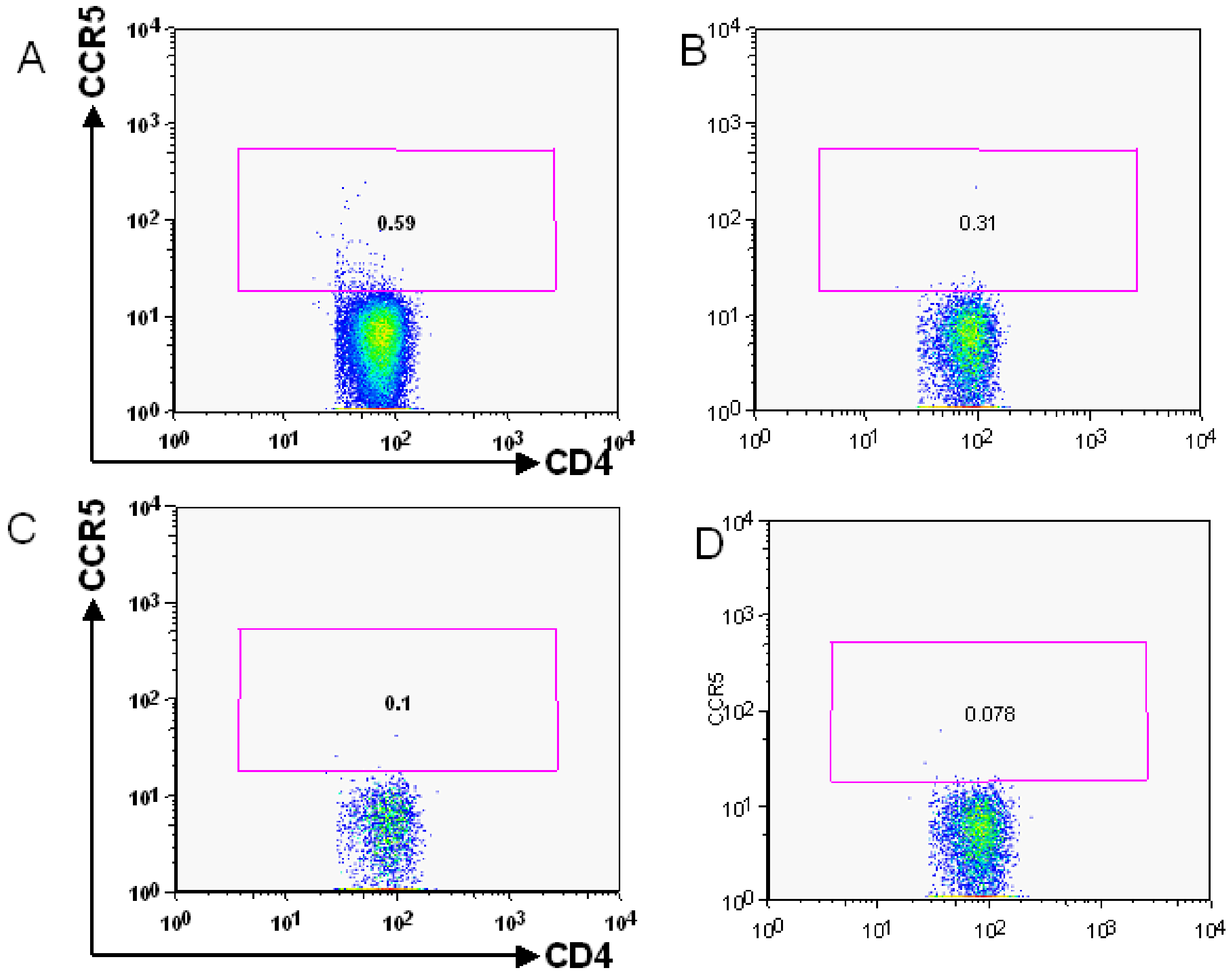

**FIGURE 2.** FACS scatter plots showing CD3+/CD4+/CCR5+ cell populations from peripheral blood mononuclear cells after incubation with the p24 peptide. Continuous decrease of the CCR5+ T-cell subpopulation with ongoing engraftment. (A) Patient before allogeneic stem cell transplantation, (B) day +61, (C) day +97, (D) day +131 after transplantation.

repeated testing of the cerebral fluid and other specimens were unsuccessful (Fig. 3). Finally, we decided to perform a neurosurgical biopsy to enforce diagnosis, which brought a new complication, namely a pneumoencephalos, with further aggravation of the patient's symptoms. Today, nearly 3 years after this event, the patient has recovered a great deal and the main residuum is the affection of the short-term memory. The nature of this event is still unclear and the results of the brain biopsy did not allow a final diagnosis.

## OPEN QUESTIONS

### Why there is no escape of X4 quasispecies?

Before receiving the stem cell transplant, the patient's HIV-1 population comprised a proportion of 2.9% X4 or dual-tropic variants. Under selective pressure by blocking CCR5 (e.g., by the CCR5 inhibitor Maraviroc), X4 is able to emerge even from proportions <1.0%[11].

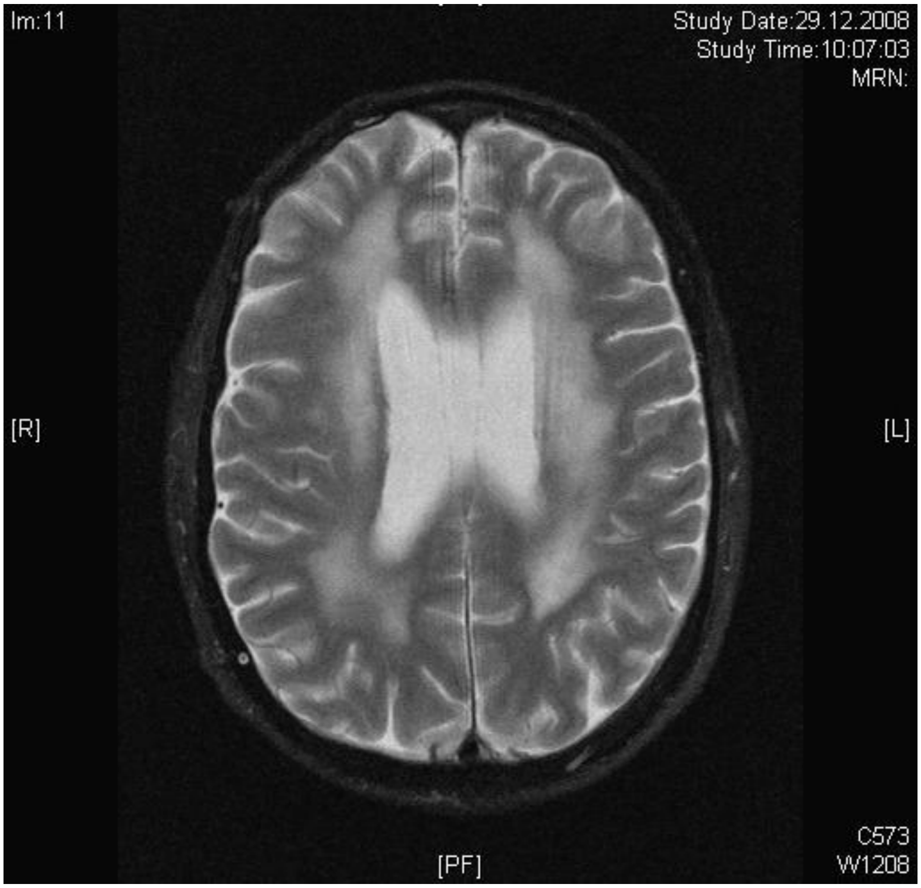

**FIGURE 3.** Patient's MRT scan on day +681 with bilateral affection of the white matter.

However, this observation may be valid for viral load in plasma, but must not necessarily reflect the situation in any other tissues or anatomical compartments. In preparation for allogeneic HSCT, most of the patient's immune cells were depleted by the conditioning regimen and it is likely that the viral pool was consequently reduced. This viral restriction was probably more efficient for X4 than for R5 viruses because X4 quasispecies are generally not able to persist in long-lasting antigen-presenting cells (APC) such as macrophages[12].

Therefore, an escape of those X4 quasispecies could have been prevented in our patient for several reasons: If not completely removed by the conditioning therapy, the number of X4-tropic infectious particles after transplantation could have been low, allowing reseeding of the patient's immune system with HIV-1. Furthermore, viral dissemination from macrophages or other APCs latently infected by either R5/X4, which had switched to an X4 genotype after the entry, seems not very likely because activation of these cell types efficiently down-regulates the CXCR4 expression on environmental T cells[13].

## Is there something special about this patient?

The natural course of HIV-1 infection is divided into three phases: (1) infection, (2) latency, and (3) CD4 depletion and consecutive death. However, some patients with HIV will stay in phase 2 for a long time without any antiretroviral therapy. These so-called long-term nonprogressors (LTNP) often have normal CD4 cell counts and a low or undetectable viral load. Currently, there are numerous potential host and viral factors described, such as the patient's HLA type or viral mutations, that may contribute to the LTNP phenomenon[14].

Concerning HLA type, the patient was KIR3DL1 homozygous and possessed *002 and *004 alleles at this locus, making him a KIR3DL1*h/*y genotype (personal communication with Nicole Bernard, Mc Gill University, Montreal, Canada). This genotype, when coexpressed with Bw4*80I and particularly with the Bw4*80I HLA allele B57, has been found to be associated with lower viral load and progression to AIDS[15]. However, the patient's HLA-B locus alleles were B7 and B35, which have no association with the KIR3DL1 allele and, in the case of B35, there is even an association with increased progression to AIDS[16].

Furthermore, although LTNP patients have low or undetectable viral levels, proviral DNA can be found regularly in blood and other tissues[17]. The absence of any detectable proviral material in our patient contradicts the thesis that a switch to an LTNP status has occurred.

## What was the role of the transplantation procedure?

The transplantation procedure consisted of a combination of chemotherapy, irradiation, and a panel of immunosuppressive medications, such as antithymocyte globulin (ATG) and cyclosporine A (CsA). All of them have been used previously in other HIV patients requiring allogeneic HSCT, and a profound and lasting change in viral replication has not been reported for any of them.

For example, ATG contains functional antibodies against T-cell markers, including CD4, CCR5, and CXCR4, resulting in a depletion of HIV-1 target cells. Recently, it has been demonstrated that ATG causes a long-lasting reduction of CD4+ T cells in HIV+ patients receiving HAART after renal transplantation[18]. However, this CD4+ depletion had no effect on the course of infection or replication of HIV-1. Nevertheless, it can be suggested that ATG is capable of reducing the viral reservoir by depletion of infected CD4+ T cells and noninfected target cells during the period of application.

CsA is a potent immunosuppressive drug commonly used in the clinical setting for immunosuppression after allogeneic transplantation. CsA has been shown to inhibit T-cell activation through a well-defined mechanism on a molecular level. The rationale for the use of CsA during primary HIV infection was to decrease the heightened state of T-cell activation observed in order to limit infection, and thus depletion, of CD4+ T lymphocytes, contributing to a better long-term preservation.

CsA was tested during primary HIV infection; CsA was not detrimental to virus-specific CD8+ or CD4+ T-cell responses. The authors suggested that rapid shutdown of T-cell activation in the early phases of primary HIV-1 infection can have long-term beneficial effects and establish a more favorable immunologic set-point. However, the activity of CsA and other immune-based therapeutic interventions during HIV infection are not sufficient to reduce the viral reservoir[19].

## Is the patient functionally cured?

The patient has been off antiretroviral medication for 4 years and there are no measurable signs of viral replication. Interestingly, the patient displayed a partial serodeconversion concerning his anti-HIV antibodies, indicating that there is no relevant virus replication left. Therefore, the possibility that the patient will suffer or die from his HIV infection is very unlikely and there is nothing else to conclude that transplantation of CCR5-delta32 stem cells has led to a functional cure.

## Is the patient sterilizing cured?

The principal idea of complete eradication of a pathogen out of the human body goes back to the Paul Ehrlichs concept of a "*therapia sterilisans magna*"[20]. In the case of HIV-1, a retrovirus that becomes part of the human genome once the individual has been infected, it is hard to imagine that all cells bearing the provirus can completely be eliminated. However, the successful experiences in cancer patients that have been cured from their malignancies by allogeneic HSCT give us some optimism that eradication could also be achieved by transplantation of HIV-resistant stem cells. The switch of patients' immune systems and bone marrow by allogeneic HSCT has led to a complete exchange of all cells derived from hematopoietic stem cells. Even all tissue-resident infected cells, like macrophages and microglia of the brain, will be replaced after a couple of times[21].

However, negative results in testing for HIV-RNA and -DNA are not sufficient to predict whether the virus will come back again. Even using new techniques for the detection of residual HIV, such as single copy assays, microdissection of tissues, or apheresis of large amounts of leukocytes from peripheral blood, will probably never solve this question as long as the results are negative.

On the other hand, viral rebound rapidly occurs after discontinuing antiretroviral therapy. In this context, a recent observation in patients with long-term antiretroviral therapy and profoundly low levels of HIV reservoir is remarkable: although one patient showed no detectable HIV-DNA both in peripheral blood cells and gut tissues, the patient developed a viral rebound 50 days after cessation of antiretroviral therapy[22,23]. In our case, during 1460 days discontinuation of antiretroviral therapy, no re-emergence of HIV was detectable, which can be interpreted in two ways: the patient achieved sterilizing cure or the patient has residual HIV-infected cells far below our current achievable limits of detection.

### Is there a Graft vs. HIV effect?

During the last decades, we have learned that the success of allogeneic HSCT in patients with malignancies is linked more to a Graft vs. leukemia/tumor effect than the intensity of the conditioning regimen[24]. In fact, even in HIV, the responses of cytotoxic T lymphocytes have an impact on the clinical course of HIV[25]. Furthermore, allogeneic stem cell transplantation itself seems to be a source of generating HIV-specific T cells with different specificity[26]. However, this proposed Graft vs. HIV effect should be independent regarding the CCR5 genotype, but to our knowledge, allogeneic HSCT alone is not sufficient to reduce the viral reservoir permanently or influence the amount of viral load.

### Why was this procedure not repeated in other patients?

After the initial publication of our report, our workgroup received several requests from other institutions concerning the possibility of finding CCR5-depleted donors for transplantation in HIV-infected patients with indication for allogeneic HSCT. Of these patients, some of them have been quite promising candidates, with large numbers of HLA-matched donors[27]. Unfortunately, in none of the donors did we find the specific homozygous CCR5-delta32 mutation. The frequency of CCR5-delta32 is connected with the ethnic background of the donors and shows a different geographical distribution[28]. Currently, there are no data of the frequency of the deletion in the group of registered stem cell donors available, except in two U.S. registries of cord blood[29,30]. Taken together, the frequency of homozygous CCR5-deleted stem cell donors will be around 1% and this will considerably reduce the probability of finding an HLA-matched donor. Despite these difficulties, our workgroup is open for requests for other patients, now and in the future.

## CONCLUSIONS

The combination of antiretroviral medication provides a long-term suppression of HIV-1 replication below the level of detection in the majority of patients. However, even sustained and efficient medication does not fully restore health and new strategies to avoid HIV-associated complications are mandatory.

Therefore, the combination of stem cell and gene-based therapies has been proposed as a long-lived alternative to antiretroviral therapy.

The case of the first successful allogeneic HSCT with CCR5-depleted stem cells in a patient with HIV infection has initiated a run towards the development of new gene therapy strategies against this disease. It is not unlikely that during the next decades, HIV treatment will enter a new level of efficiency and will move us one step closer towards a cure.

## ACKNOWLEDGMENTS

We thank Kate Krauss and Stephen LeBlanc from the AIDS Policy Project and Jeffrey Laurence, Senior Scientific Consultant for programs at amfAR, for supporting our work.
